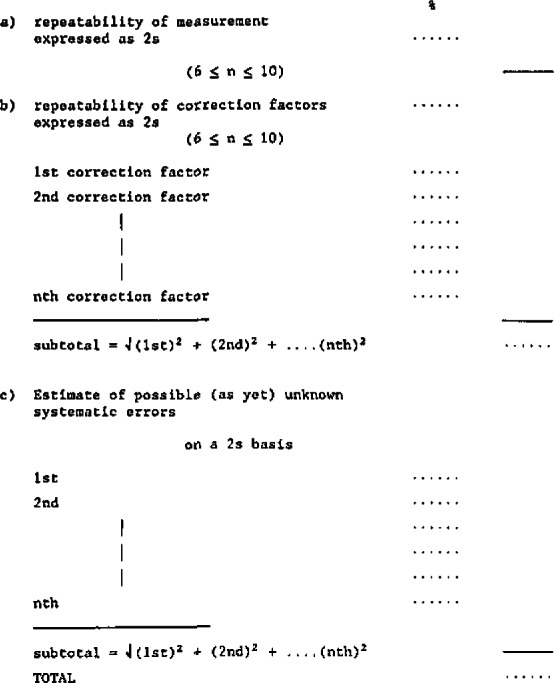# Transferring Accuracy to the Trace Level and Then to the Field

**DOI:** 10.6028/jres.093.142

**Published:** 1988-06-01

**Authors:** Paul De Biévre

**Affiliations:** Central Bureau of Nuclear Measurements, Commission of the European Communities—JRC, B-2440 Geel, Belgium

The problem of arriving at an “accurate” determination in trace analysis—determining a “true” small amount in a large amount—requires the assessment of the performance of the measurement method used to an appropriate detail.

In the case of “direct” measurement techniques of trace concentrations, i.e., assaying directly a small amount relative to the large matrix sample, the evaluation should include the assessment of the reproducibility of the linear, or non-linear response of the measurement chain and of the high sensitivity detector over many orders of magnitude down to small concentrations. (See example in fig. 1.) To allow this assessment, a set of synthetic isotope mixtures can be prepared gravimetrically and used to examine the measurement chain and detector. An example of such a set is given in table 1. Its preparation is described elsewhere [1]. The built-in ratio ^235^U/^238^U ≈ 1 (certified to 0.02%) can be used to determine any systematic errors in the “excitation” or “ion” source and correct the observed ^233^U/^235^U or ^233^U/^238^U ratios. Comparison of the latter to the certified values (given to ±0.03%) then provides a means to determine systematic errors of the (sensitive) detectors used in the trace analysis (e.g., electron multipliers) and to determine possible deviations from linearity of the (electronic) measurement equipment used. It is also possible to determine the reproducibility of these systematic errors and/or deviations allowing to correct for them. It is important to point out that the uncertainties of such corrections must be carried on and correctly propagated until the final result in any measurement of unknowns.

We now have a means for specialized measurement laboratories to verify how their detector and measuring equipment is really performing at (very) low signals, i.e., at the trace element level and a first example obtained is given in figure 2.

In the case of measurement techniques which assay small amounts in weighed samples, the “jump” to the trace level is performed through weighing (by using small weights) and the problem becomes one of assaying a trace amount with a known, proven, or provable accuracy.

Every indication is there that this will have to come from isotope-specific—or should I say nuclide-specific?—methods on the basis of the fact that isotopes are very specific representatives of elements and specificity is an essential key to accuracy.

We have at present a few nuclide-specific assay methods in our array of analytical assay methods:

- Those which use the property of one nuclide as in a specific nuclear reaction (“activation analysis”); the degree of specificity will influence heavily the potential for accuracy; the degree of quantitative knowledge of every parameter concerned will determine the size of the total “inaccuracy.”
- Those which modify an isotope (nuclide) abundance ratio *R*_x_ in the sample (“isotope dilution”), by the addition of a “spike” which is a known number of atoms of the same element as the unknown but with another R_y_ value for the abundance ratio of the same isotopes (an “enriched” stable isotope- or “nuclide”); this spike acts as an almost ideal “internal standard” and the measurement is reduced to the determination of the new isotope abundance ratio *R*_B_ in the blend resulting from the change induced by *R*_y_ in *R*_x_ Of course, proper isotopic homogenization and complete destruction of the matrix (this makes the assay matrix—independent) in a closed system, is an absolute prerequisite for this type of assay.

Isotope- or nuclide-specific methods do have inherently more potential for accuracy since they measure isotopes and not elements. Isotopic atoms are measured on the basis of properties of the atom nucleus (e.g., difference in mass) which are shielded from chemical interferences before or during the measurement, by identical outer electron clouds. They can therefore potentially lead to greater accuracy (when the isotopic measurement methods are correctly applied of course). It is to be expected that isotope- or nuclide-specific methods will more and more deliver reference values and methods for elemental trace analysis in the future.

Thus the measurement of an element concentration ratio (= conventional analytical chemistry) is replaced by the measurement of isotope abundance ratio. The essential step of isotopic homogenization can be guaranteed when properly carried out, because it takes advantage of the fact that the outer electron clouds (i.e., the chemical form) of both sample and spike isotopic atoms are identical. This ensures that both will behave in the same way and end up in the same chemical forms after chemical destruction of the sample. At the moment of full isotopic homogenization, the end result is in fact “frozen in.” Any chemical effect in a later stage of the process, e.g., losses in normal purification or separation stages, will therefore affect equally the isotopes of sample and spike in the same way and leave their abundance ratio—the end result—unchanged.

The “accurate” values obtained as explained above for trace elements in unknown samples, can now be carried to the field by Interlaboratory Measurement Evaluation Programmes (IMEPs) where measurements on these samples are collected and graphically displayed around the “reference value.” One could also call such a programme an “external” measurement evaluation.

Current work at the Central Bureau for Nuclear Measurements (CBNM) aims at following the route described above to establish reference values for IMEPs. An example of what this yields is given in figure 3, taken from the nuclear field: even after suitable calibration with available Reference Materials, a picture develops showing considerably larger spread than what laboratories believe to be their uncertainty. It is a surprisingly classical picture which can be produced for any assay measurement in any field at any concentration level. Only the ordinates and the title of the picture must be changed, the general nature of the picture remains the same (see an example in fig. 4). In other words, the concept is expected also to be applicable to the trace analysis field.

The question arises where such “reference value” or “baseline” takes its authority from and why. Since it is very difficult to unequivocally establish these baseline values, this should be done by specialized institutes or laboratories (the “standards” laboratories in the world) along the following lines:

- It must be absolutely transparent how the value intended to be the “closest approximation of the true value” (i.e., intended to be accurate), is arrived at, based on a detailed explanation of each step in the characterization process which leads from our bank SI units to the value pretending to be able to serve as “reference,” and
- It must be absolutely transparent how the “uncertainty” or “inaccuracy” of the value is arrived at, based on the establishment of a list of uncertainty contributors which are identified qualitatively and individually quantified by various appropriate procedures so that their component contribution to the total “inaccuracy” is clearly visible; a work model for that is given in table 2.

Note that such IMEP programmes are result-oriented and not method- or procedure-oriented. In the graphs presenting the results, distinction is clearly made between:

- The state of the practice (SOP) which is materialized in the spread of the results and in the (non-)deviation from the established reference values.
- Same form of the state of the art (SOA) which is materialized in the uncertainty level of the “reference values” which must be demonstrated to be reliable enough and usable for such “external” QA programmes.

## Conclusions

1. It has been shown how the response of measuring equipment can be evaluated for (non-) linearity over many orders of magnitude, this possible error contributor being separated from the contributors located in the “source” or “excitation” part of the measurement method.
2. Isotope- or nuclide-specific methods have a great potential to serve “reference” purposes so badly needed in trace analysis.
3. IMEPs with carefully established baselines should carry references to the field.
4. “Standards-” or “Reference-” Institutes should consider it as part of their basic mission to deliver regularly “baseline-values” for real life samples which are circulated as “blind” samples amongst the laboratories wanting to assess their true measurement capability through an “external” evaluation programme.

## Figures and Tables

**Figure 1:**
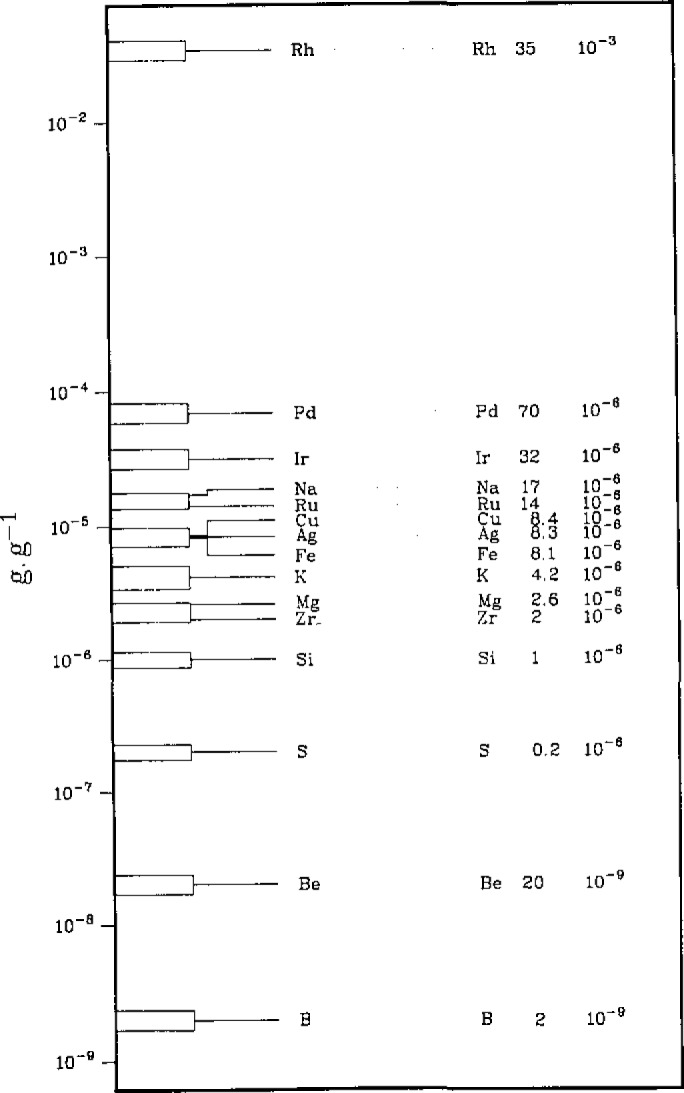
Example of dynamic range of trace concentrations: impurities in Pt metal (Spark Source Mass Spectrometry).

**Figure 2:**
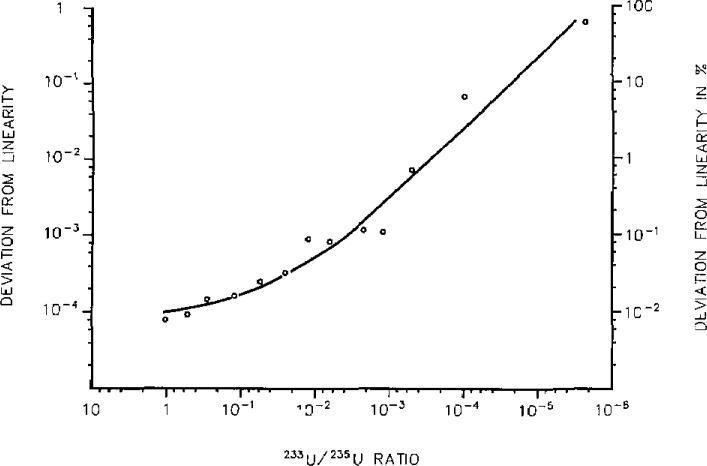
Test of the response of a mass spectrometer as function of the ratio to be measured.

**Figure 3:**
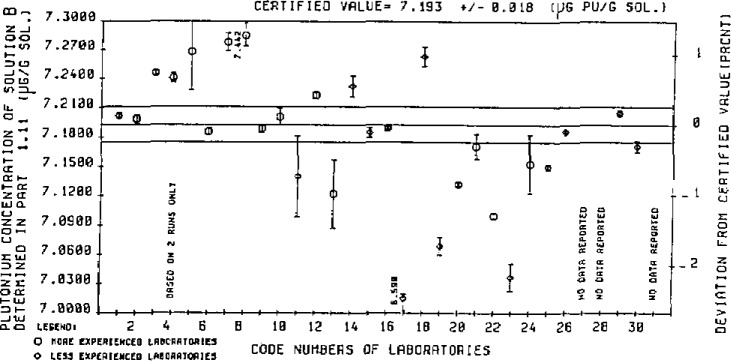
Interlaboratory measurement evaluation programme on Pu assay amongst 31 laboratories (Reference Value: CBNM by IDMS).

**Figure 4:**
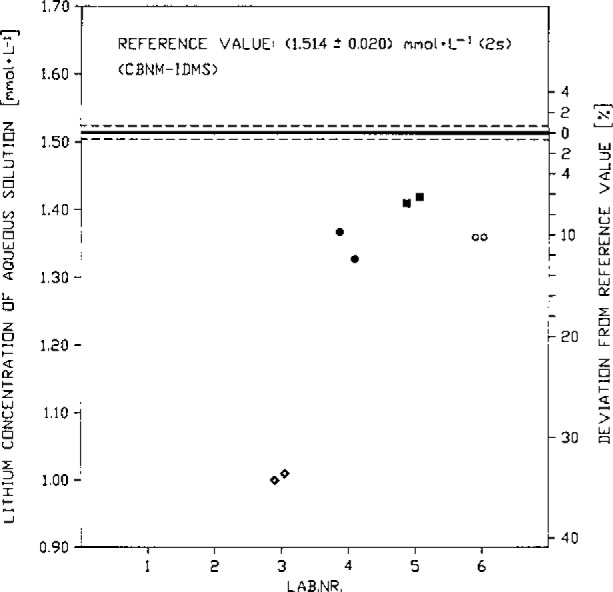
Interlaboratory measurement evaluation programme on Li assay amongst 61 laboratories (Reference Value: CBNM by IDMS).

**Table 1:** Set of (isotopic) reference materials to test the linearity of a detector/amplifier measurement system

Code number	Description	Atomic isotope ratios		Unit
		^233^U/^235^U ±0.03% of value	^235^U/^238^U ±0.000 30	
CBNM-IRM-				
072/1	Uranyl-nitrate solution	1.000 33	0.991 03	1 mg U in 1 g solution
072/2		0.699 67	0.991 68	
072/3		0.499 85	0.992 12	
072/4		0.299 87	0.992 56	
072/5		0.100 01	0.992 99	
072/6		0.050 091	0.993 10	
072/7		0.019 994	0.993 17	
072/8		0.010 165	0.993 19	
072/9		0.005 000 0	0.993 20	
072/10		0.002 001 2	0.993 21	
072/11		0.000 968 92	0.993 21	
072/12		0.000 500 88	0.993 21	
072/13		0.000 101 82	0.993 21	
072/14		0.000 019 996	0.993 21	
072/15		0.000 001 999 5	0.993 21	

**Table 2:** Proposed Work Model to Establish a “Composition of Uncertainty”